# Quantitative Assessment of Fundus Tessellated Density and Associated Factors in Fundus Images Using Artificial Intelligence

**DOI:** 10.1167/tvst.10.9.23

**Published:** 2021-08-18

**Authors:** Lei Shao, Qing Lin Zhang, Teng Fei Long, Li Dong, Chuan Zhang, Wen Da Zhou, Ya Xing Wang, Wen Bin Wei

**Affiliations:** 1 Beijing Tongren Eye Center, Beijing Key Laboratory of Intraocular Tumor Diagnosis and Treatment, Beijing Ophthalmology & Visual Sciences Key Lab, Medical Artificial Intelligence Research and Verification Key Laboratory of the Ministry of Industry and Information Technology, Beijing Tongren Hospital, Capital Medical University, Beijing, China; 2 Department of Neurosurgery, Tsinghua University Yuquan Hospital, Beijing, China; 3 Aerospace Information Research Institute, Chinese Academy of Sciences (CAS), Beijing, China; 4 Beijing Institute of Ophthalmology, Beijing Ophthalmology & Visual Science Key Lab, Beijing Tongren Eye Center, Beijing Tongren Hospital, Capital Medical University, Beijing, China

**Keywords:** deep learning, computer-aided labeling, fundus tessellated density, subfoveal choroidal thickness, fundus photography, fundus image

## Abstract

**Purpose:**

This study aimed to quantitative assess the fundus tessellated density (FTD) and associated factors on the basis of fundus photographs using artificial intelligence.

**Methods:**

A detailed examination of 3468 individuals was performed. The proposed method for FTD measurements consists of image preprocessing, sample labeling, deep learning segmentation model, and FTD calculation. Fundus tessellation was extracted as region of interest and then the FTD could be obtained by calculating the average exposed choroid area of per unit area of fundus. Besides, univariate and multivariate linear regression analysis have been conducted for the statistical analysis.

**Results:**

The mean FTD was 0.14 ± 0.08 (median, 0.13; range, 0–0.39). In multivariate analysis, FTD was significantly (*P* < 0.001) associated with thinner subfoveal choroidal thickness, longer axial length, larger parapapillary atrophy, older age, male sex and lower body mass index. Correlation analysis suggested that the FTD increased by 33.1% (*r* = 0.33, *P* < .001) for each decade of life. Besides, correlation analysis indicated the negative correlation between FTD and spherical equivalent (SE) in the myopia participants (*r* = −0.25, *P* < 0.001), and no correlations between FTD and SE in the hypermetropia and emmetropic participants.

**Conclusions:**

It is feasible and efficient to extract FTD information from fundus images by artificial intelligence–based imaging processing. FTD can be widely used in population screening as a new quantitative biomarker for the thickness of the subfoveal choroid. The association between FTD with pathological myopia and lower visual acuity warrants further investigation.

**Translational Relevance:**

Artificial intelligence can extract valuable clinical biomarkers from fundus images and assist in population screening.

## Introduction

Fundus tessellation, defined as the visibility of large choroidal vessels at the posterior fundus pole outside of the peripapillary region, is the only simple way to observe the choroidal vascular structure under direct vision.^1^^,^^2^^–^^5^ Previous studies have found that fundus tessellation is closely related to age and myopic refractive error and has been treated as one of the important incipient manifestations of pathological myopia.^3^^–^^5^ Besides, fundus tessellation may also be associated with various ocular diseases, such as angle closure glaucoma, age-related macular degeneration (AMD), pathological myopia, central serous chorioretinopathy, choroidal neovascularization, and uvitis, among others.^1^^,^^6^^–^^14^ All the factors that affect the visibility of choroidal vessels can be reflected in the fundus tessellation qualitatively, including the atrophy of choroidal capillaries, the density of choroidal pigment, the distribution of choroidal vessels, and more.^6^ Although morphological characteristics of fundus tessellation could be observed visually, traditional imaging analysis are limited in measurement accuracy.^1^^,^^15^ Because of technical limitations, it is impossible to quantitatively extract effective indicators of fundus tessellation from abnormal fundus images for analysis.^7^ Recently, with the development of artificial intelligence image processing technology, computer vision and region of interest (ROI) extraction can effectively and efficiently identify the texture nuance that cannot be distinguished by human eyes.^8^ Artificial intelligence, involved convolutional neural network and computer vision, developed to implements the computational systems to extract representations directly from huge numbers of images without designing explicit hand-crafted features.^16^^,^^17^ The applications of artificial intelligence techniques trained on fundus images can automatically detect various ophthalmic diseases with competitive or close-to-expert performance.^18^^,^^19^ This study assesses the distribution of fundus tessellated density (FTD) and its associations with other ocular and systemic factors and ocular diseases by population-based epidemiological research, which establishes a new quantitative index of fundus tessellation on the basis of artificial intelligence image processing technology simply to extract the exposed choroidal area on the fundus image.

## Methods

### Study Population

The Beijing Eye Study is a population-based study conducted in northern China. It was performed in five communities in the urban district of Haidian in northern central Beijing and in three communities in the village area in the Daxing district south of Beijing. An age of no less than 50 years was the only inclusion criterion. The study has been described in detail.^10^^,^^11^ The Medical Ethics Committee of Beijing Tongren Hospital approved the study protocol, and all participants gave informed consent according to the Declaration of Helsinki. The current study population was derived in 2011, when participants were invited for the second five-year follow-up examination, at which time the enhanced depth imaging spectral-domain optical coherence tomography was performed on the participants that can acquire choroidal information. Of a total population of 4403 individuals aged ≥ 50 years, 3468 (response rate, 78.8%) individuals (1963 female, 56.6%) participated in the examinations. The study was divided into a rural part (1633 subjects, 47.1%; 943 female, 57.7%) and an urban part (1835 subjects, 52.9%; 1020 female, 55.6%). The mean age was 64.6 ± 9.8 years (median, 64 years; range, 50–93 years).

### Ophthalmic and General Examinations

All examinations were carried out in schoolhouses or common houses of the eight communities included. Trained research technicians asked questions of the study participants from a standardized questionnaire on demographic variables such as age, gender, level of education, occupation, eye disease and systemic disease history, lifestyle, cognitive function, depression, and more. After obtaining informed consent, fasting blood samples were taken for measurement of blood lipids, creatinine, hemoglobin, C-reactive protein, and glucose. Blood pressure and body height and weight were measured and recorded. The ophthalmic examination included measurement of presenting visual acuity, uncorrected visual acuity, and best corrected visual acuity, slit lamp–assisted biomicroscopy of the anterior segment of the eye, biometry applying optical low-coherence reflectometry (Lensstar 900; Optical Biometer, Koeniz, Switzerland), and fundus photographs (nonstereoscopic photograph of 45° of the central fundus; fundus camera type CR6-45NM; Canon Inc., Tokyo, Japan).

Automatic refractometry (Auto Refractometer AR-610; Nidek Ltd, Tokyo, Japan) was performed on all the participants. If uncorrected visual acuity was 1.0 (i.e., 5/5), subjective refractometry was also performed. The spherical equivalent (SE) was calculated according to the format: SE = spherical degrees + (cylindrical degrees/2). Myopia was defined if SE was < −0.25 D; hypermetropia was defined if SE was > +0.25 D. All the participants with undilated pupils were imaged with a Heidelberg Spectralis (Heidelberg Engineering, Heidelberg, Germany; wavelength: 870 nm; scan pattern: enhanced depth imaging) with the instrument positioned close enough to the eye to produce an inverted image.

### Extraction and Quantification of FTD by Artificial Intelligence

**Figure 1.**
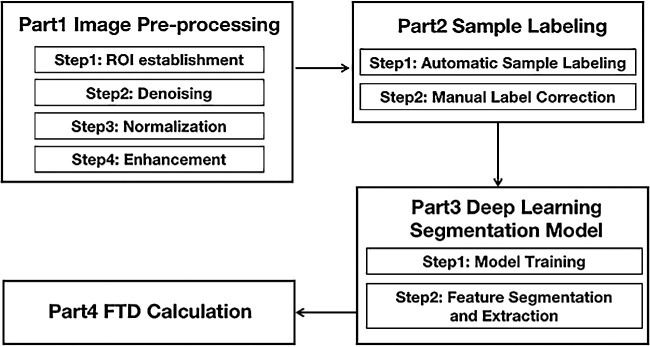
The flow chart of the proposed algorithm for FTD calculation.

In this study, we extracted the exposed choroid from the fundus through artificial intelligence based image processing technology, then calculated the average exposed choroid area of per unit area of fundus, named it FTD. Figure 1 indicates the flow chart of the proposed algorithm. The process of obtaining FTD is composed of preprocessing, sample labeling, deep learning segmentation model, and FTD calculation. The image preprocessing (Fig. 3) involves four steps: ROI establishment, denoising, normalization, and enhancement. The sample labeling is a semiautomatic part including automatic sample labeling and manual label correction. The deep learning segmentation model consists of model training and feature segmentation and extraction. The process of calculating FTD includes the following parts:

#### Image Preprocessing

• ROI establishment: ROI establishment is to extract the effective area in the fundus image and remove the invalid areas such as the background, then the interference with the exposed choroid extraction been reduced. We first perform channel separation of the color fundus image, where the background area is dark in the red channel. And then use the threshold segmentation method to segment the red channel image to obtain the ROI candidate area by the average gray value and the area ratio of the dark area. Finally, we screen the ROI candidate area by the morphological features and its location, then ROI is established.
• Denoising: Denoising is to reduce noise interference during shooting and imaging. We realize it by low-pass filtering method, which converts the image from the spatial domain to the frequency domain. The denoising can be achieved by removing the low-frequency part.
• Normalization: We adjust the color, brightness and size of each image to a uniform range through average calibration, to reduce the difference between images and the deviation of brightness and color. The brightness normalization is achieved by converting the image from color space to LAB space, calibrating the average value of the L space, and the transfer back to the RGB space.
• Enhancement: In the ROI area, we use the Contrast-Limited Adaptive Histogram Equalization (CLAHE) algorithm to enhance the image. It divides the image into different small blocks, and performs gray-scale restriction enhancement processing on each small block, then performs gray-scale interpolation between adjacent small blocks to eliminate the gray-scale difference between the boundaries of small blocks.

#### Sample Labeling

Sample labeling includes two parts: automatic sample labeling and manual label correction, which is semi-automatic. Automatic labeling obtained by channel separation of color fundus images and channel subtraction. After that, we manually correct the samples obtained by automatic labeling. And then we produce the final sample image.

#### Deep Learning Segmentation Model

• Model Training: We use the labeled samples as training samples, based on the deep learning semantic segmentation network (ResnetFCN) training model, first extract high-level features through Resnet18, and then deconvolution to obtain the segmentation area, output the leopard spot confidence map, and obtained the confidence probability of each pixel on the fundus belongs to exposed choroid, and finally the exposed choroid of fundus was obtained through threshold segmentation.
• Feature segmentation and extraction: Finally, we use the trained model to extract the exposed choroidal area (Fig. 4).

#### FTD Calculation

Based on the extracted choroidal exposed area, we calculate the average choroidal exposed area of per unit area on fundus to obtain FDT(ρ). ρ = S1/S, where *S1* is the extracted area of the exposed choroid and *S* is the area of the ROI obtained by preprocessing.

### Performance Evaluation of Segmentation Model

To test the performance of the segmentation model, three general indicators of accuracy, sensitivity, and specificity have been calculated to evaluate the results of model. The accuracy was 0.9652, the sensitivity was 0.7247, and the specificity was 0.9605.

### Statistical Analysis

Statistical analysis was performed using a commercially available statistical software package (SPSS for Windows, v. 26.0; IBM-SPSS, Chicago, IL, USA). The data of all the right eyes was included into the current analysis. First, we examined the mean values (presented as mean ± standard deviation) of FTD. Variance analysis was applied to compare the difference of the FTD among the different age groups. Second, we performed a univariate linear regression analysis with FTD as a dependent parameter while ocular and systemic parameters as independent parameters. Pearson correlation and multiple regression analysis were demonstrated for variation in FTD relative to age and refractive error. Third, we performed a multivariate linear regression analysis using the stepwise method. FTD defined as a dependent parameter, and the explanatory variables that were significantly associated with FTD in univariate analysis been appropriately selected as independent parameters. All *P* values were two-sided and considered statistically significant when the values were <0.05; 95% confidence intervals were presented.

## Results

### Baseline Characteristics

Among 3468 eyes of 3468 participants, FTD was available for 3074 individuals (88.6%) (1733 female [56.4%]). There was no FTD measurements for the eyes with opacities of the optic media due to no fundus photographs could be taken or the insufficient quality of the images. Any ocular disease, involved lesions of the optic nerve or macula, was no reason to be excluded if the quality of fundus image was assessable. The mean age was 64.1 ± 9.7 years (median, 63 years; range, 50–93 years); the mean SE was −0.13 ± 1.96 D (median, 0.25 D; range, −20.0 to 13.50 D); the mean axial length was 23.22 ± 1.09 mm (median, 23.13 mm; range, 18.96–30.88 mm). Compared with the group of subjects with and without FTD calculation, the group without measurement was significantly (*P* < 0.001) older (68.6 ± 9.8 years vs. 64.1 ± 9.7 years), more likely have myopic (−1.06 ± 3.75 D vs. −0.13 ± 1.96 D) and longer axial length (23.66 ± 1.57 mm vs. 23.22 ± 1.09 mm), but did not vary significantly in gender (*P* = 0.45).

### Univariate and Multivariate Analysis of FTD

The mean FTD was 0.14 ± 0.08 (median, 0.13; range, 0–0.39). Table 1 shows the correlations between FTD and systemic or ocular parameters in univariate analysis.

**Table 1.** Univariate Analysis of Associations Between Fundus Tessellated Density and Ocular and Systemic Parameters in the Beijing Eye Study 2011

Parameter	Regression Coefficient B	Standardized Coefficient Beta	95% CI	*P* Value
Systemic parameters				
Age	0.00	0.33	0.00 to 0.00	<0.001
Gender	−0.02	−0.13	−0.028 to −0.016	<0.001
Height	0.00	0.06	0.00 to 0.001	0.002
Weight	−0.00	−0.08	−0.001 to 0.00	<0.001
Body mass index	−8.86	−0.95	−8.965 to −8.748	<0.001
Rural region of habitation	0.01	0.12	0.007 to 0.012	<0.001
Diastolic blood pressure	−0.00	−0.10	−0.001 to 0.000	<0.001
History of cerebral infarction	0.01	0.06	0.003 to 0.012	0.001
Serum concentrations of hemoglobin	−0.01	−0.06	−0.009 to −0.002	0.003
Serum concentrations of creatinine	0.000	0.11	0.000 to 0.000	<0.001
Serum concentrations of C-reactive protein	0.88	0.96	0.864 to 0.892	<0.001
Serum concentrations of glucose	−0.00	−0.07	−0.005 to −0.001	<0.001
Smoking	3.55	0.97	3.518 to 3.590	<0.001
Alcohol consumption	−36.11	−39.87	−37.880 to −34.329	<0.001
Depression	−9.40	−0.91	−9.572 to −9.230	<0.001
History of cardiovascular disease				0.540
History of myocardial infarction				0.92
Ocular perfusion pressure				0.06
Serum concentrations of cholesterol				0.08
Aspirin intake				0.28
Snoring				0.41
Systolic blood pressure				0.50
Serum concentrations of high-density lipoproteins				0.48
Serum concentrations of low-density lipoproteins				0.11
Serum concentrations of triglycerides				0.93
Presence of diabetes mellitus				0.06
Ocular parameters				
Axial length	0.03	0.40	0.03 to 0.03	<0.001
Refractive error	−0.01	−0.25	−0.01 to −0.01	<0.001
Anterior chamber depth	0.02	0.12	0.01 to 0.03	<0.001
Lens thickness	0.02	0.09	0.01 to 0.03	<0.001
Steeper cornea	0.05	0.14	0.03 to 0.06	<0.045
Best-corrected visual acuity	−0.07	−0.17	−0.08 to −0.05	<0.001
Central corneal thickness	−0.46	−0.98	−0.46 to −0.46	<0.001
Subfoveal choroidal thickness	−0.66	−0.83	−0.68 to −0.64	<0.001
Optic disc area	2.57	0.13	1.47 to 3.68	<0.001
Retinal nerve fiber layer thickness	−2.40	−0.96	−2.44 to −2.37	<0.001
Retinal vein obstruction	22.89	0.13	15.83 to 29.96	<0.001
Intermediate AMD	3.53	0.09	2.07 to 4.98	<0.001
Late AMD	18.28	0.24	15.40 to 21.15	<0.001
Parapapillary atrophy area	−0.56	−0.68	−0.58 to −0.54	<0.001
Corneal diameter				0.78
Prevalence of diabetic retinopathy				0.21
Early AMD				0.39
Prevalence of geographic atrophy of the macula				0.75
Degree of cortical cataract				0.54
Subcapsular cataract				0.15
Open-angle glaucoma				0.95
Angle-closure glaucoma				0.10

In the multivariate analysis, we firstly take the FTD as a dependent variable, and all significantly associated parameters (*P* < 0.05) as independent variables after adjustment for age. Secondly, we dropped those parameters indicating a high degree of collinearity which include height, weight, anterior chamber depth, lens thickness (the variance inflation factor was greater than 3); or no longer significantly associated with the FTD (*P* > 0.05), involving the diastolic blood pressure (*P* = 0.94), rural region of habitation (*P* = 0.85), history of cerebral infarction (*P* = 0.27), serum concentrations of hemoglobin (*P* = 0.54), creatinine (*P* = 0.10), C-reactive protein (*P* = 0.25), and glucose (*P* = 0.52), smoking (*P* = 0.50), alcohol consumption (*P* = 0.55), depression (*P* = 0.32), refractive error (*P* = 0.65), steeper cornea (*P* = 0.08), best corrected visual acuity (*P* = 0.97), central corneal thickness (*P* = 0.89), optic disc area (*P* = 0.88), retinal nerve fiber layer thickness (*P* = 0.09), retinal vein obstruction(*P* = 0.55), intermediate AMD (*P* = 0.43), late AMD (*P* = 0.70).

The final model (correlation coefficient *r* = 0.74) shows FTD was significantly associated with thinner subfoveal choroidal thickness (SFCT), longer axial length, larger parapapillary atrophy, older age, male sex, and lower body mass index (*P* < 0.001). (Table 2).

**Table 2.** Multivariate Analysis of Associations Between Fundus Tessellated Density and Ocular and Systemic Parameters in the Beijing Eye Study 2011

Parameter	Regression Coefficient B	Standardized Coefficient Beta	95% CI	Variance Inflation Factor	*P* Value
Systemic parameters					
Age	0.08	0.08	0.00 to 0.00	1.29	<0.001
Gender	−0.02	−0.11	−0.02 to −0.01	1.11	<0.001
Body mass index	−0.00	−0.06	−0.00 to 0.00	1.04	<0.001
Ocular parameters					
Axial length	0.01	0.14	0.01 to 0.01	1.35	<0.001
Parapapillary atrophy	0.01	0.09	0.00 to 0.00	1.29	<0.001
Subfoveal choroidal thickness	0.00	−0.58	−0.00 to 0.00	1.35	<0.001

CI, confidence interval.

**Figure 2.**
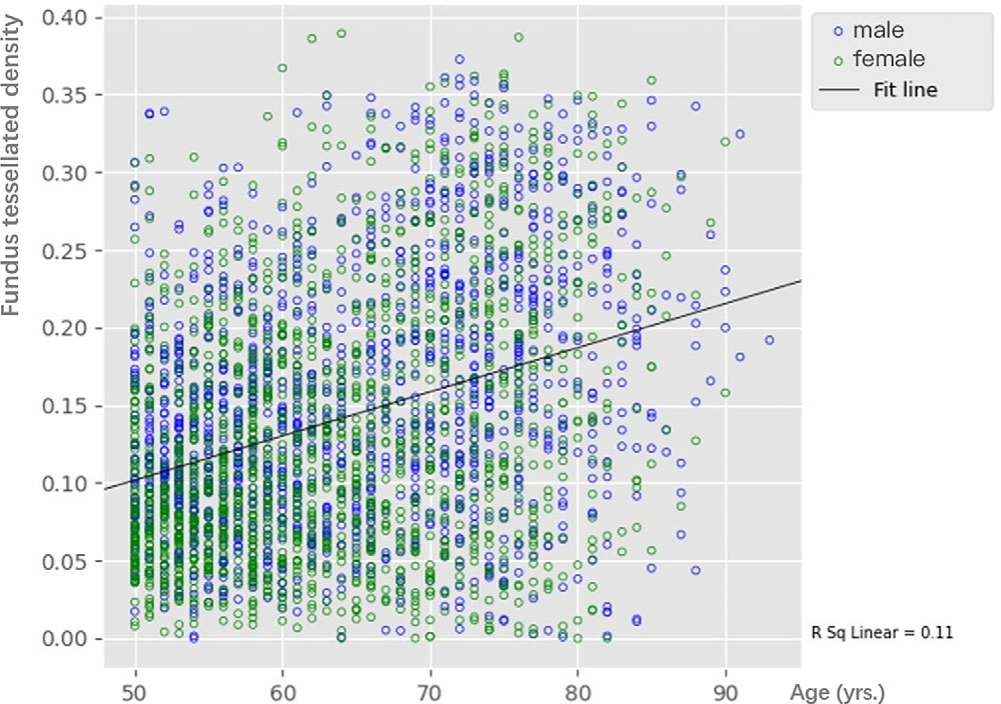
Scatter dots plot of the association between l fundus tessellated density and age in the elderly Chinese population aged 50 or more.

The FTD was increased with aging (*F* = 179.71, *P* < 0.001) (Table 3). If the whole study population was stratified into age groups of 10 years each, correlation analysis showed the FTD is positively correlated with age (*r* = 0.33, *P* < 0.001) (Fig. 2). Regression analysis suggested that the FTD increased by 33.1% for each decade of life. The FTD of male (0.15 ± 0.08) was significantly greater than females (0.13 ± 0.08) (*t* = 7.12, *P* < 0.001) (Table 3).

**Table 3.** Fundus Tessellated Density in Different Age Groups in the Elderly Chinese Population Aged 50 or More

	N	Mean Fundus Tessellated Density	SD	*P* Value
Total	3074	0.14	0.08	
Age				<0.001
50+	1210	0.12	0.06	
60+	882	0.14	0.08	
70+	982	0.18	0.09	
Gender				<0.001
Male	1341	0.15	0.08	
Female	1733	0.13	0.08	

**Figure 3.**
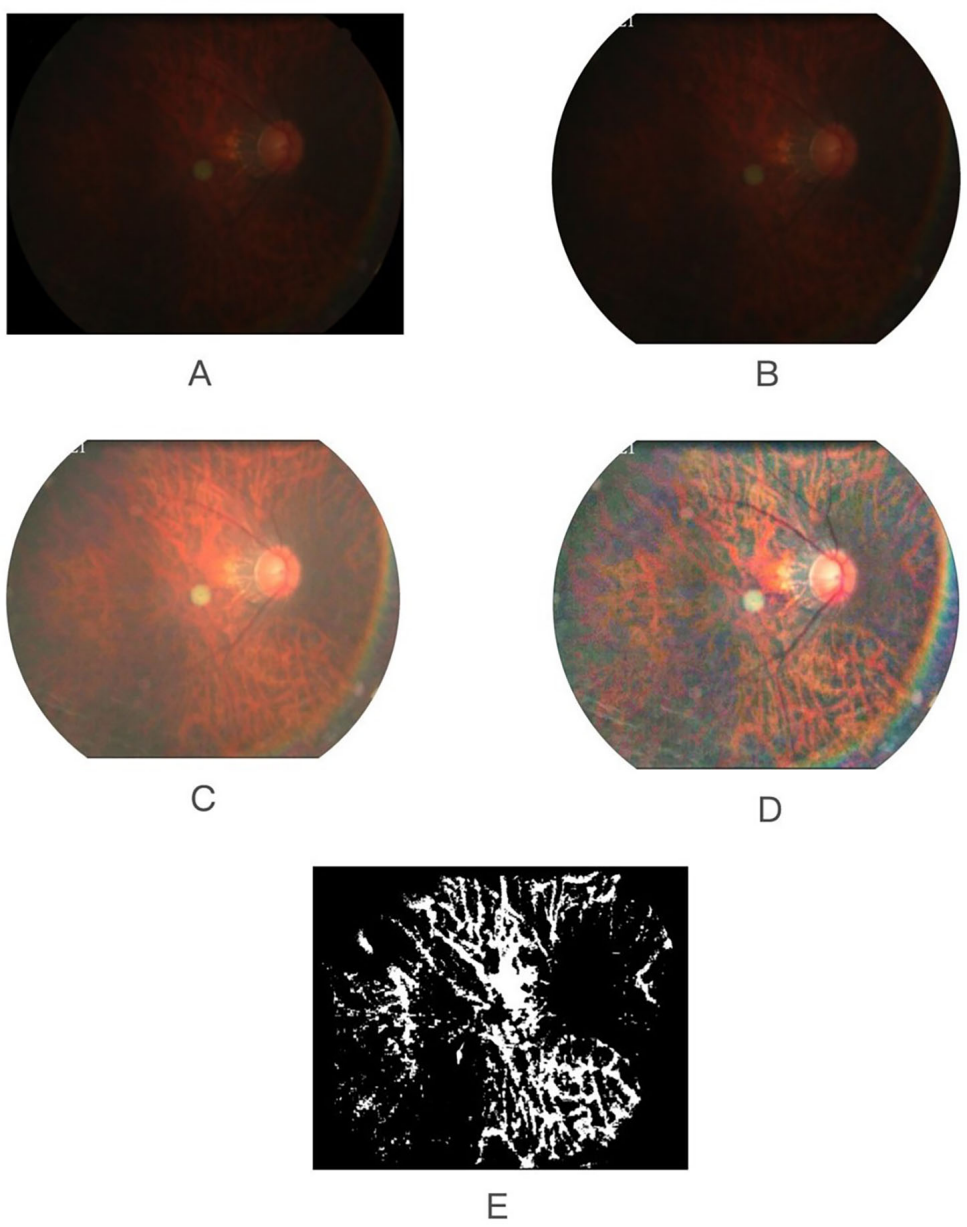
Extraction of fundus tessellated density by artificial Intelligence technology. (**A**) Original image. (**B**) ROI. (**C**) The image after de-drying and normalization. (**D**) The enhanced image. (**E**) The exposed choroidal annotation image based on the enhanced image.

**Figure 4.**
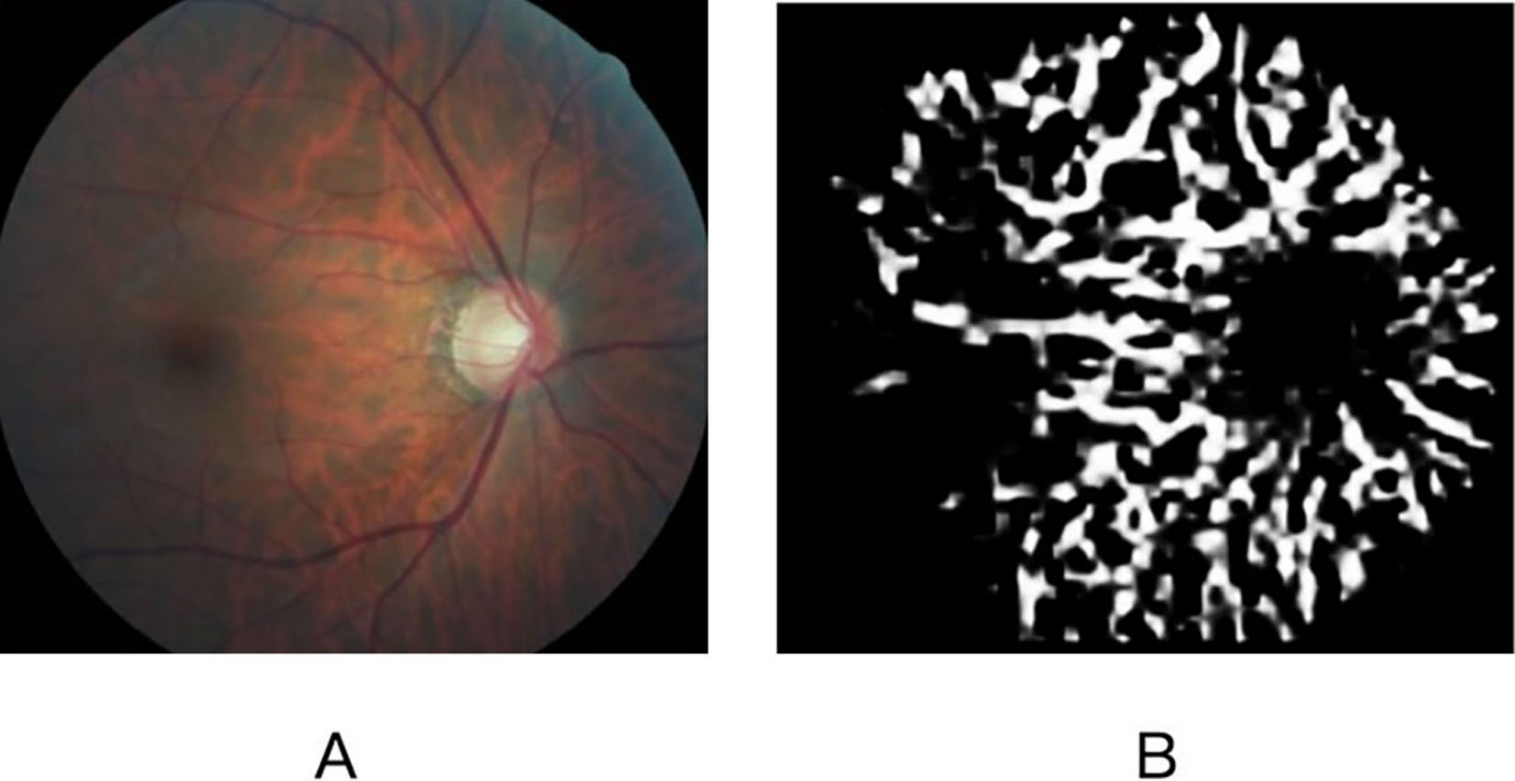
Quantification of fundus tessellated density by artificial Intelligence technology. (**A**) Original image. (**B**) The exposed choroidal image extracted automatically.

The regressions of the associations of FTD with axial length or FTD with refractive error showed a curvilinear course. Correlation analysis showed there was negative correlation between FTD and SE in the whole participants (*r* = −0.25, *P* < 0.001). For the myopia population, the mean SE was −2.16 ± 2.40 D. Correlation analysis showed there was negative correlation between FTD with SE in the myopia participants (*r* = −0.25, *P* < 0.001). There were no correlations between SE and FTD in the hypermetropia and emmetropic participants (*P* > 0.05), as shown in Table 4.

**Table 4.** The Fundus Tessellated Density in the Different Refractive Status

Refractive Error	N	Mean Fundus Tessellated Density	SD	Mean SE	SD SE	*R*	*P* Value
Emmetropic	259	0.13	0.08	0.00	0.00	0.05	0.50
Hypermetropia	1669	0.13	0.08	0.94	0.97	−0.04	0.14
Myopia	1146	0.16	0.09	−2.16	2.40	−0.25	<0.001

## Discussion

To our knowledge, this study is the first quantitative measurements study of FTD from color photographs through artificial intelligence image processing technology. There were qualitative or semiquantitative studies on FTD in the past.^12^^,^^15^^,^^20^^–^^22^ Spaide^20^ reported that OCT examination showed fundus tessellation in 28 cases of choroidal thinning; Yoshihara and colleagues^21^ observed 100 cases with an average age of 25.8 ± 3.9 years and found that the degree of fundus tessellation was significantly correlated with the thickness of subfoveal choroid. The high correlation between fundus tessellation and choroidal thickness has also been confirmed by hospital-based clinical studies, including AMD, glaucoma, and high myopia patients^12^^,^^15^^,^^22^ Parallel to our study, with a population-based recruitment of participants and a relatively large sample extends the findings of the previous mostly hospital-based investigations, the FTD was not associated with open-angle (*P* = 0.95) or angle-closure glaucoma (*P* = 0.10). Although the FTD is related to intermediate (b = 0.09, *P* < 0.001) or late (b = 0.24, *P* < 0.001) AMD in univariate analysis, the multivariate model to remove the variable of AMDs and SFCT remain the most significant association.

The advantage of this study is that it is the first quantitative study of FTD by latest artificial intelligence extraction quantitative technology. Thus there is no comparable study to assess the precision of FTD. In addition, the sample size of this study is large. Compared with previous studies, our results are consistent with a series of previous qualitative or semiquantitative studies; that is, FTD can be used as a quantitative biomarker to evaluate the choroidal thickness in the general population. In the large-scale epidemiological investigation, we can extract the FTD information from the fundus images of people with different ages and diopters to evaluate SFCT and screen people with abnormal SFCT preliminarily. In high myopia patients, we can quantitatively evaluate the changes of SFCT through regular follow-up of FTD. With higher popularity in screening, FTD obtained by color fundus image may be expected to replace SFCT (measured by EDI-OCT) as a new quantitative index for choroidal analysis.

In our population-based study, we found that mean FTD was 0.14 ± 0.08, ranging from 0 to 0.39. In the multivariate analysis, FTD was significantly greater in elderly male individuals with lower body mass index. The ocular parameters with strong statistically associations involved thinner SFCT, longer axial length, and larger parapapillary atrophy (*P* < 0.001).

Several studies, including the current study, found consistently that the fundus tessellation was aggravated significantly with aging.^1^^,^^2^^,^^20^ In our recent quantitative assessment, the mean FTD was 0.12 ± 0.06 of the 50–59 age group; 0.14 ± 0.08 of the 60–69 age group; 0.13 ± 0.08 of the more than 70 age group. Regression analysis suggested that the FTD increased by 33.1% for each decade of life. It revealed that the FTD may be used as a visual indicator of aging.

Besides SFCT and age, the present multivariate analysis also showed that FTD was significantly associated with longer axial length and larger parapapillary atrophy, which considered as important indexes of early phase of myopic development.^5^^,^^23^^,^^24^ Further stratified analysis showed the negative correlation between FTD and SE in the myopia participants; but the correlation was not found in the hypermetropic and emmetropic participants (Table 4). And the FTD of myopia population (0.16 ± 0.09) was significantly (*P* < 0.001) larger than hypermetropic (0.13 ± 0.08) and emmetropic groups (0.13 ± 0.08). On account of the larger quantity of sample in our proposed research, the association between FTD and the SE performed more significantly than other studies.^11^^,^^24^^,^^25^ The associations can be explained by the atrophy of choroid in the myopic eye, especially in the high myopic eyes.

Potential limitations should be mentioned. First, differences between participants and nonparticipants may have led to a selection artifact with a reasonable response rate of 78.8% in the Beijing Eye Study 2011. Second, the FTD was assessed only in the right eye of each individual, so that inter-eye differences could not be analyze. Third, it is important to assess the reproducibility for a new technology of quantitative measurement. In a recent separate study, the FTD measurements showed a repeatability for 10 reexaminations with an intraclass coefficient of 1.00 (own data). Forth, our investigation included all eligible subjects from the study region for the population-based study. Thus cases with diseases may have affected the FTD, especially either in relation to choroidal thickening or thinning.

In conclusion, the elderly Chinese population has an FTD with a mean of 0.14 ± 0.08. After adjusting for ocular and systemic parameters, FTD is also related to thinner SFCT, longer axial length, larger parapapillary atrophy, older age, male sex, and lower body mass index. Because color fundus imaging has high popularity in screening and is easy to assess, FTD may be expected to replace SFCT (measured by EDI-OCT) as a novel quantitative index for choroidal analysis. Its association with pathological myopia and lower visual acuity warrants further investigation.
